# Radiation Therapy for Breast Cancer With Oligometastatic Cervical Lymph Nodes: A Case Report

**DOI:** 10.7759/cureus.51617

**Published:** 2024-01-03

**Authors:** Hiroshi Burioka, Unta Yamamori, Natsuko Nagano, Atsushi Ue, Yukihisa Tamaki

**Affiliations:** 1 Radiation Oncology, Shimane University Faculty of Medicine, Izumo, JPN

**Keywords:** multimodality treatment, cervical lymph node, oligometastases, radiation therapy, breast cancer

## Abstract

Stage IV breast cancer is difficult to cure and is mainly treated with systemic therapy. However, when distant metastasis is oligometastatic, proactive treatment including local therapies for the primary lesion and distant metastases has been reported to improve prognosis. We encountered a patient who had left breast cancer with ipsilateral cervical lymph node metastases. The metastases were oligometastatic, and we treated them curatively. The patient was a female in her 50s who had been aware of a lump in the lower inner quadrant of the left breast for a few years. A biopsy was performed and left breast cancer was diagnosed pathologically. Radiological examination showed metastasis to ipsilateral axillary and cervical lymph nodes. The cervical lymph node metastases were oligometastatic, suggesting possible improvement in prognosis by multimodality treatment including local therapy. The multimodality treatment in this case comprised mastectomy with levels I and II axillary lymph node dissection, systemic therapy (including chemotherapy, endocrine therapy, and molecular targeted therapy), and postmastectomy radiation therapy. The left chest wall and left supraclavicular lymph node region were irradiated. Furthermore, following the postmastectomy radiation therapy, the cervical lymph node metastases were treated with radical radiation therapy. The cure was achieved, with recurrence-free status maintained for two years and four months after the completion of radiation therapy. This case suggests that, for breast cancer with oligometastatic involvement of cervical lymph nodes, locally treating these distant metastatic lesions with radical radiation therapy as part of multimodality treatment is beneficial.

## Introduction

Hellman and Weichselbaum proposed a definition of oligometastases [1]. They attributed the oligometastatic state to limited metastatic potential, which implies that aggressive local therapy in combination with systemic therapy could potentially achieve a cure. The Clinical Practice Guidelines for Breast Cancer in Japan define oligometastatic disease as when the number of distant metastasis sites is small (usually three to five or less) [2]. Breast cancer with distant metastasis is classified as stage IV by the Union for International Cancer Control (eighth edition). Stage IV breast cancer is difficult to cure, and systemic therapy is the mainstay of treatment [3]. However, a number of studies have reported that local therapies, such as surgery and radiation therapy, lead to longer survival and cure in patients with oligometastatic breast cancer [2,4,5]. However, in those patients, the locally treated oligometastatic sites were common sites of distant metastasis in breast cancer (e.g., bone, lungs, liver, and brain) [6]. Distant metastasis to cervical lymph nodes is rare in patients with breast cancer, and the treatment in such cases has not been established [7,8]. Here, we report a rare case of oligometastatic breast cancer that spread to distant cervical lymph nodes, in which cure was achieved by multimodality treatment including systemic therapy in combination with local therapy for both the primary site and cervical lymph node metastases. In addition, the relevant literature is also discussed in this study.

## Case presentation

**Figure 1:**
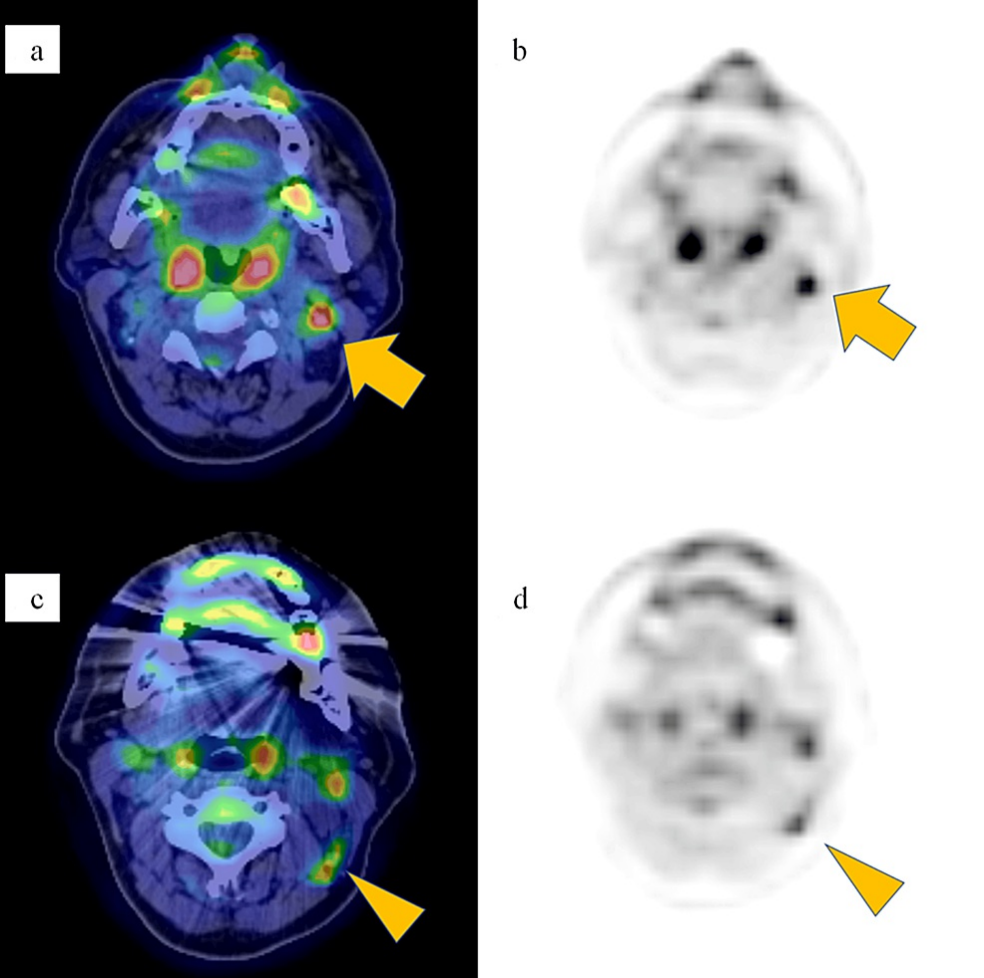
Radiological findings at the initial examination. (a, b) An ^18^F-fluorodeoxyglucose-position emission tomography (FDG-PET) image in the delayed phase/computed tomography, a fusion image acquired in the axial plane, and an FDG-PET image in the delayed phase acquired in the axial plane show ^18^F-fluorodeoxyglucose (FDG) accumulation (standardized uptake value max of 5.3 in the early phase and 6.6 in the delayed phase) at a cervical lymph node (arrow). (c, d) An FDG-PET image in the delayed phase/computed tomography, a fusion image acquired in the axial plane, and an FDG-PET image in the delayed phase acquired in the axial plane show FDG accumulation (standardized uptake value max of 4.1 in the early phase and 4.2 in the delayed phase) at a cervical lymph node (arrowhead).

The patient was a female in her 50s who had been aware of a lump in the lower inner quadrant of the left breast for a few years. A biopsy was performed, and left breast cancer was diagnosed pathologically. Radiological examination showed metastasis to ipsilateral axillary nodes. Further, ^18^F-fluorodeoxyglucose positron emission tomography showed ^18^F-fluorodeoxyglucose accumulation at two cervical lymph nodes (short axis, 7 mm and 5 mm) at level II (defined by Robbins et al.) on the left side, indicating distant metastases (Figures 1a-1d) [9]. The ^18^F-fluorodeoxyglucose accumulation in breast cancer increases for about 3 hours, whereas the ^18^F-fluorodeoxyglucose accumulation in inflammatory lesions decreases after 1 hour [10,11]. Therefore, we clinically diagnosed the left cervical lymph nodes as the metastatic lymph nodes, not the lymph nodes affected by the inflammation of the head and neck (submandibular region, etc.) based on the ^18^F-fluorodeoxyglucose positron emission tomography findings, although we don’t have a pathological diagnosis of the left cervical lymph nodes. Taken together, the diagnosis was stage IV breast cancer (cT3N2aM1) according to the Union for International Cancer Control (eighth edition). Because of the oligometastatic nature of the ipsilateral cervical lymph node involvement, we opted for local therapies for the primary site and ipsilateral cervical lymph node metastases in combination with systemic therapy, in the hope of improving the prognosis. Thus, the treatment strategy for this patient included systemic therapy, surgery, adjuvant radiation therapy for the primary site, and radical radiation therapy for the ipsilateral cervical lymph node metastases.

**Figure 2:**
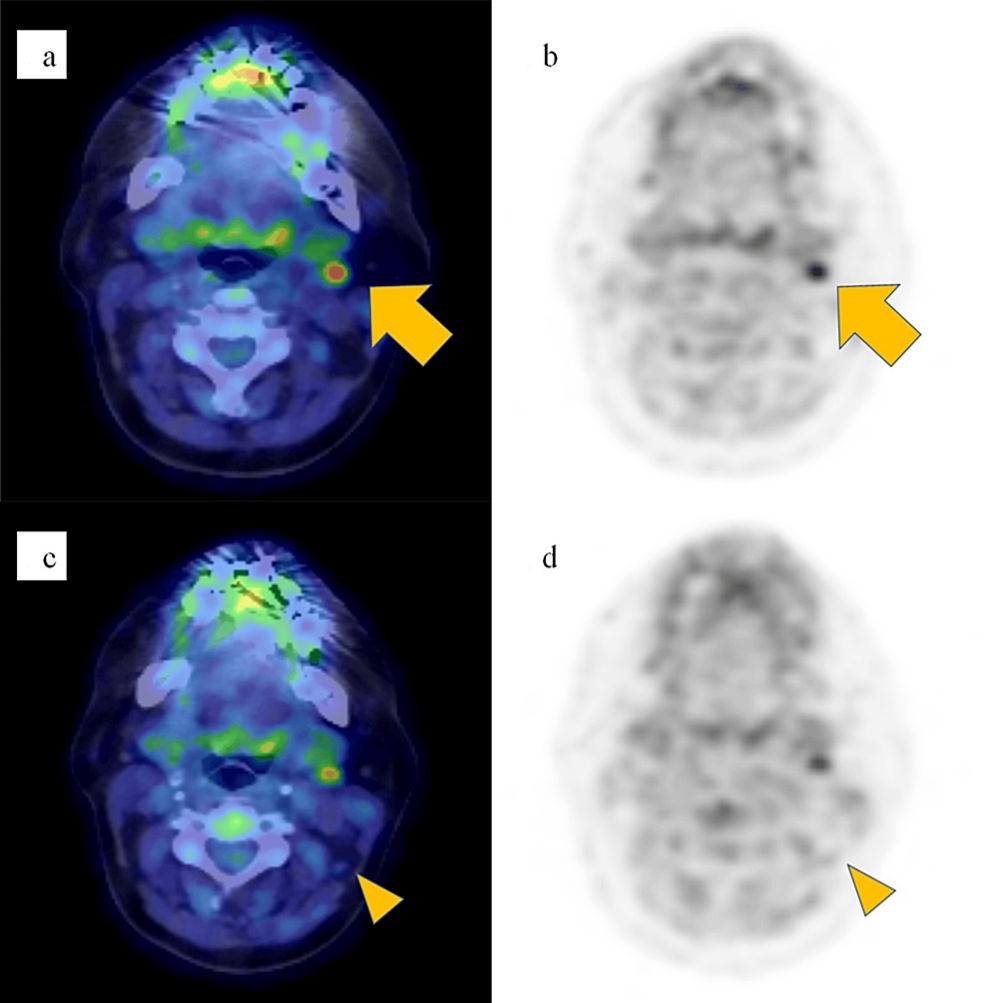
Radiological findings about four months after the start of chemotherapy. (a, b) An ^18^F-fluorodeoxyglucose-position emission tomography (FDG-PET) image in the delayed phase/computed tomography, a fusion image acquired in the axial plane, and an FDG-PET image in the delayed phase acquired in the axial plane show reductions in size and FDG accumulation (standardized uptake value max of 3.8 in the early phase, and 5.2 in the delayed phase) compared with the initial examination at a cervical lymph node (arrow). (c, d) An FDG-PET image in the delayed phase/computed tomography, a fusion image acquired in the axial plane, and an FDG-PET image in the delayed phase acquired in the axial plane show reductions in the size of a lymph node with almost complete disappearance of FDG accumulation (arrowhead).

She received the following treatments between 2020 and 2021 at Shimane University Hospital and Hyakudomi Clinic, Izumo, Shimane, Japan. First, mastectomy with levels I and II axillary lymph node dissection was performed. Invasive ductal carcinoma (pT2N3a) was diagnosed pathologically. The tumor was estrogen receptor-positive and progesterone receptor-positive, but HER2-negative. Second, systemic therapy with cytotoxic anticancer drugs was administered that comprised a total of four cycles of dose-dense epirubicin and cyclophosphamide and a total of four cycles of dose-dense paclitaxel. After chemotherapy, the short axes of the two lymph nodes at level II on the left side decreased to 4 mm and 2 mm, leading to judge that chemotherapy for the cervical lymph node metastases was effective (Figures 2a-2d).

**Figure 3:**
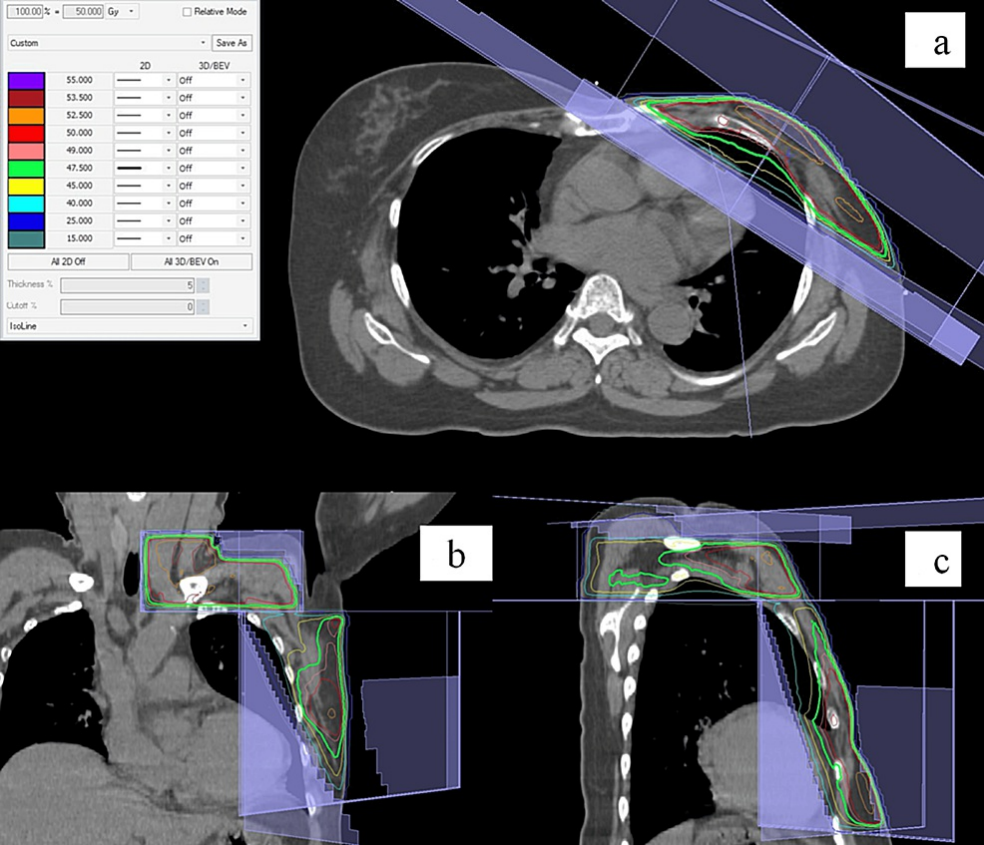
Dose distribution of postmastectomy radiation therapy. The green line indicates the 95% isodose line. (a) Dose distribution on an axial image. (b) Dose distribution on a coronal image. (c) Dose distribution on a sagittal image.

**Figure 4:**
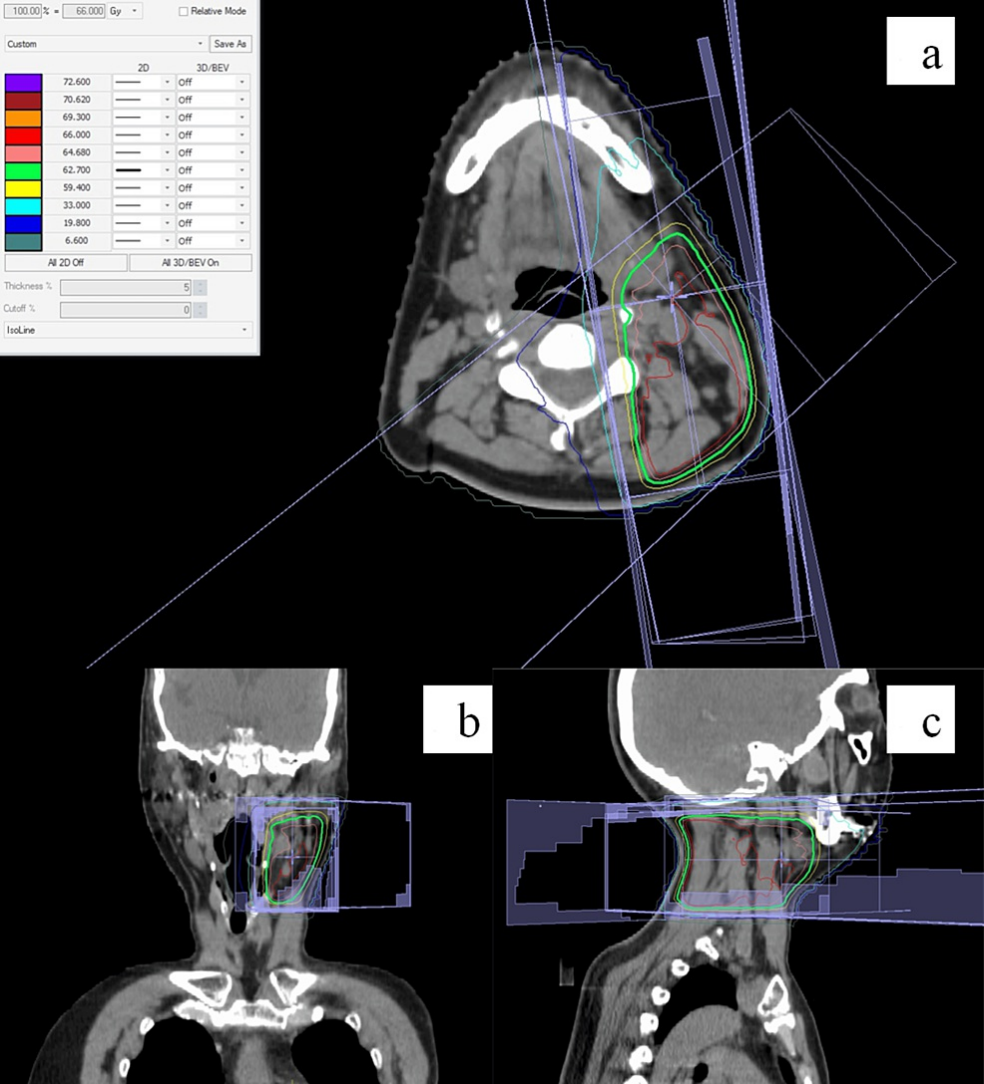
Dose distributions in radiation therapy to the cervical lymph node metastases and to ipsilateral Robbins levels II to III region. The green line indicates a 95% isodose line. (a) Dose distribution on an axial image. (b) Dose distribution on a coronal image. (c) Dose distribution on a sagittal image.

Third, systemic therapy was given that comprised endocrine therapy with letrozole and molecular-targeted therapy with palbociclib. Finally, radiation therapy was administered. First, postmastectomy radiation therapy (50 Gy in 25 fractions) was administered to the left chest wall, the left supraclavicular lymph node region, and the left infraclavicular lymph node region (Figures 3a-3c). Then, radical radiation therapy, using a radiation dose for head and neck cancers, was administered to the cervical lymph node metastases (Figures 4a-4c). In the radiation therapy, radiation was applied to the region covering the cervical lymph node metastases and the entire cervical lymph node level II (as defined by Robbins et al.) on the left side (50 Gy in 25 fractions), and then, a shrinking field targeting only the cervical lymph node metastases was irradiated (16 Gy in 8 fractions) [9]. The total radiation dose for radical radiation therapy was 66 Gy.

**Figure 5:**
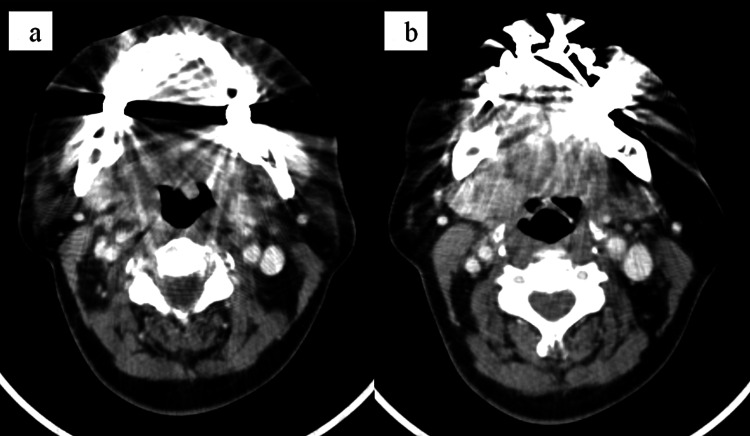
Radiological findings about two years and two months after the start of radiation therapy. (a, b) The axial computed tomography images. The cervical lymph node metastases had shrunk and were no longer identifiable.

In all three-dimensional conformal radiation therapy sessions, 6-MV x-rays were used. Stereotactic radiotherapy for cervical lesions was considered, but we chose three-dimensional conformal radiation therapy due to safety concerns and the lack of consensus on the optimal radiation dose for stereotactic radiotherapy. Palbociclib was withdrawn during radiation therapy. All radiation therapies were completed successfully. Adverse events were assessed using the Common Terminology Criteria for Adverse Events (version 5.0). Acute adverse events of radiation therapy were grade 2 radiation dermatitis in the left cervical region, grade 2 alopecia at the left back of the neck, and grade 1 esophagitis, all of which resolved shortly after the completion of radiation therapy. Grade 1 lymphedema occurred as a late adverse event, but had resolved by one year and four months after the completion of radiation therapy. As of this writing, recurrence-free status has been maintained for two years and four months after the completion of radiation therapy, and we are still following up on this patient (Figures 5a, 5b).

## Discussion

In breast cancer, common sites of distant metastasis include bone, lungs, liver, and brain [6]. Metastasis of breast cancer to cervical lymph nodes is rare, occurring in approximately 1% of patients [7]. In addition, Salama et al. reported that among breast cancer patients with distant metastasis, approximately 50% and 75% had ≤2 and ≤4 metastatic lesions, respectively, suggesting that the proportion of oligometastases is by no means low [4].

Dorn et al. reported that the prognosis of oligometastases was better than that of multiple distant metastases in patients with breast cancer [12]. In that study, a comparison between patients with spread of breast cancer to ≤5 distant sites and those with spread to >6 distant sites revealed median overall survival of 107.7 months and 22 months, respectively, after failed chemotherapy and five-year actuarial survival rates of 59.6% and 11.6%, respectively, showing that prognosis was markedly better in the former group than in the latter group. Thus, oligometastatic patients whose cancer has spread to a small number of distant sites require treatment strategies different from the conventional strategy that involves systemic therapy only.

At the St. Gallen International Breast Cancer Consensus Conference in 2021, multimodality treatment including curative therapy was supported for treating breast cancer with 1-2 oligometastatic sites [13]. Local therapies such as radiation therapy and surgery have been used to treat oligometastases of breast cancer, but there are no established therapies [2,4,5].

Radiation therapy including stereotactic radiotherapy for lung or liver oligometastases from breast cancer showed two- and four-year overall survival rates of 76-95% and 59%, respectively, and two-year and four-year progression-free survival rates of 44-53% and 38%, respectively, suggesting oligometastases can be cured by local therapy using radiation therapy [14,15]. However, in those reports, oligometastases were observed in the bone, lungs, liver, and brain, and cervical lymph node metastases were not mentioned in any of the previous reports.

Lin et al. reported that multimodality treatment combining surgery, chemotherapy, and radiation therapy conferred a significant survival advantage to patients with breast cancer who had synchronous isolated distant lymph node metastasis, including cervical lymph node metastasis [16]. In that study, overall survival was comparable between patients with synchronous isolated distant lymph node metastasis (M1) and those with N3cM0 stage disease. Taking together the findings reported by Lin et al. and the recommendations from the St. Gallen International Breast Cancer Consensus Conference in 2021, we believe that proactively providing multimodality treatment including local therapy is significant in treating breast cancer with oligometastatic involvement of cervical lymph nodes [16].

In the present study, we assessed whether three-dimensional conformal radiation therapy (standard fractionated radiotherapy) or stereotactic radiotherapy (hypofractionated radiotherapy) should be used for cervical lymph node metastases in multimodality treatment. Given that carotid blowout occurred after stereotactic radiotherapy for neck oligometastases in patients with a history of prior radiotherapy to the neck and we couldn’t reach a consensus on the optimal radiation dose for stereotactic radiotherapy for cervical lesions, three-dimensional conformal radiation therapy was chosen after assessing the risks and benefits in this case [17-19]. As of two years and four months after the completion of radiation therapy, late adverse events, including carotid blowout, have not occurred in the neck region.

Our case report has some limitations. First, we don’t have a pathological diagnosis of the left cervical lymph nodes because we didn’t perform a biopsy on the cervical lymph nodes. Second, we diagnosed the left cervical lymph node metastases based on the ^18^F-fluorodeoxyglucose positron emission tomography findings. We can’t completely exclude the possibility that the inflammation of the head and neck (mandible, etc.) caused the ^18^F-fluorodeoxyglucose accumulation because we don’t have a pathological diagnosis of the left cervical lymph nodes. Finally, cervical lymph node metastasis in breast cancer is defined as stage IV by the Union for International Cancer Control (eighth edition). On the other hand, Kim et al. reported that the prognosis of patients with cervical lymph node metastasis was similar to that of patients with supraclavicular lymph node metastasis in breast cancer [20]. There is a possibility that the prognosis of patients with cervical lymph node oligometastasis is better than that of patients with other oligometastatic sites (bone, lungs, liver, brain, etc.) in breast cancer. Randomized controlled trials are needed to clarify this.

## Conclusions

The cure was achieved by multimodality treatment including radiation therapy in this case, suggesting that, for breast cancer with oligometastatic involvement of cervical lymph nodes, local therapy for cervical lymph node metastases, in combination with systemic therapy and local therapy for the primary site, is beneficial. This case demonstrates that proactive addition of local therapy to treat local metastasis lesions, if oligometastatic, can lead to improved prognosis. However, reports on such patients with oligometastatic breast cancer are limited, and the accumulation of more cases and randomized controlled trials are awaited to gain new insights.
